# Agar/Carboxymethyl Cellulose Blended Films with Green-Synthesised Silver Nanoparticles as a Sustainable Alternative for Food Packaging Applications

**DOI:** 10.3390/polym17233126

**Published:** 2025-11-25

**Authors:** Seyedeh Fatemeh Mirpoor, Alessio Massironi, Danielle Winning, Stella Lignou, Sameer Khalil Ghawi, Federico Trotta, Dimitris Charalampopoulos

**Affiliations:** 1 Department of Food and Nutritional Sciences, University of Reading, Harry Nursten Building, Pepper Lane, Whiteknights, Reading RG6 6DZ, UK; 2 Metalchemy Limited, 71-75 Shelton Street, London WC2H 9JQ, UK

**Keywords:** polysaccharide-based film, active packaging, silver nanoparticles, green chemistry, blended film, bionanocomposite, sustainable packaging

## Abstract

The shelf life of food can be affected by storage and transport conditions. The development of a biodegradable, eco-friendly active bioplastic for food packaging could delay food deterioration during these stages, while minimising the environmental impact of non-degradable conventional plastics. In this study, blended films of agar with carboxymethyl cellulose (CMC) were integrated with different concentrations of silver nanoparticles (AgNPs) that were produced by a green synthesis method. The incorporation of silver nanoparticles into the blended films increased the stiffness of the film and improved the water vapour barrier and hydrophobicity. The thermal stability and the Fourier transform infrared spectra of the films were not affected by the different concentrations of AgNPs incorporated. The film microstructure was affected by the concentration of AgNPs and resulted in an increase in the film’s pore size. Films with the highest concentration of AgNPs showed antibacterial activity against foodborne pathogens, *L. monocytogenes*, *Staphylococcus aureus*, *Pseudomonas aeruginosa* and *E. coli*, and provided the material with the highest UV protection and bio-disintegration in soil and simulated seawater environments compared to the other developed films. The developed agar/CMC blended films with improved physicochemical properties present a viable alternative to conventional plastics in active food packaging applications.

## 1. Introduction

The modern lifestyle has extensively increased the demand for ready-to-eat food and fresh-cut fruits and vegetables [1]. However, many of these foods are very sensitive to spoilage and deterioration, leading to substantial amounts of food waste on a daily basis, at a time when many people worldwide suffer from hunger and malnutrition [2]. Globally, approximately USD 1.2 trillion’s worth of food is wasted each year due to deterioration, equating to about one third of all food produced for human consumption [3]. In this context, developing active food packaging presents a promising solution for extending food shelf life and reducing food waste [4].

To this end, nanomaterials have been widely used as additives in packaging materials, significantly improving their physicochemical properties, such as strengthening mechanical properties, enhancing barrier properties and introducing effective antibacterial and antioxidative properties [5]. Importantly, the antimicrobial activity of these nanomaterials has the potential to delay or prevent microbial growth in the packed food [6,7]. Silver nanoparticles (AgNPs) have gained significant attention in recent years for their role in active packaging production. This is due to their low cytotoxicity, antimicrobial activity against a wide range of bacteria and the ability to extend the shelf life of packed foods [8,9,10].

In recent years, AgNPs have been produced by several physical, chemical and biological methods. The most common method is chemical synthesis, which involves the use of several toxic chemicals and reducing agents [11]. Physical methods are faster and do not involve the use of chemicals, but they have higher energy requirements [12]. On the other hand, eco-friendly biological or green methods utilise plant extracts, microorganisms and enzymes. These methods boast several advantages over conventional methods, including the absence of toxic chemicals, low energy consumption and easy recovery [10,12,13]. Conventional plastics possess excellent physicochemical properties, can be produced at low cost (two–four times cheaper than bioplastics) and have a wide range of applications. These characteristics of plastics have resulted in their worldwide use, but consequently, their disposal has led to serious environmental pollution, where an estimated 300,000 tonnes of plastic particles are now contaminating the world’s oceans [14].

Moreover, most traditional plastics are made of a combination of polymers, making them difficult to recycle. In contrast, the production of bioplastics, made from biopolymers, that can be easily degraded in the environment without generating toxic microplastics, is a promising alternative [15]. Polysaccharides are types of biopolymers widely utilised in the development of bioplastics due to their outstanding physicochemical properties and biodegradability [9,16].

One of the most commonly used polysaccharides for the production of bioplastics is agar, a linear polysaccharide made from β(1,3)- and α(1,4)-linked galactose. Agar is extracted from red seaweeds and has extensive applications in the food industry as a thickening and clarifying agent.

Agar is a biodegradable, biocompatible and edible polymer with good water resistance; however, low thermal stability and brittleness limit its application in food packaging [17]. On the other hand, carboxymethyl cellulose (CMC), an anionic cellulose derivative, is non-toxic, biodegradable and relatively inexpensive. It forms transparent and flexible films, but exhibits high water sensitivity due to the large number of hydroxyl and carboxyl groups [18]. Therefore, blending agar with another polysaccharide, like CMC, is a potential method to minimise their individual drawbacks [19]. Zhao et al. (2022) report on the production of agar-based films blended with gelatin, gellan gum and κ-carrageenan, which were modified with cellulose nanocrystals and calcium chloride. This modification improves the physicochemical properties of the produced bioplastics, extending the shelf life of strawberries [20]. Macieja et al. (2022) also report the development of an active carboxymethylcellulose-based film incorporated with silver nanoparticles reduced by melanin. They further investigated the properties of the film, which exhibited higher antimicrobial activity at higher concentrations of silver nanoparticles [21].

This work aims to investigate the effect of incorporating green-synthesised silver nanoparticles on the physicochemical and antimicrobial properties of a blended bioplastic consisting of agar and CMC, designed for the food packaging sector. To this end, the optimum blending conditions of agar/CMC and the concentration of silver nanoparticles were investigated. The developed films were characterised according to various physicochemical properties, such as antimicrobial activity, water sensitivity, bio-disintegration and mechanical, thermal and optical properties.

## 2. Materials and Methods

### 2.1. Materials

Agar, carboxymethyl cellulose (CMC), sodium hydroxide, hydrochloric acid, and glycerol (GLY) were purchased from Sigma-Aldrich (Manchester, UK). The G-AgNPs (green-synthesised AgNPs in aqueous suspension) were formulated in-house at Metalchemy’s laboratory (London, UK). Mueller–Hinton agar, nutrient agar and tryptic soy agar were supplied by Thermo Scientific™ (Loughborough, UK). *Escherichia coli* ATCC 25922, *Listeria monocytogenes* 1043S, *Staphylococcus aureus* NCTC 8532 and *Pseudomonas aerujinosa* NCTC 10322 were obtained from the Department of Food and Nutritional Sciences at the University of Reading and were used to perform the antimicrobial assays.

### 2.2. Preparation and Casting of the Film-Forming Solutions

The stock solution of CMC (2% *w*/*v*) was prepared by dispersing CMC in distilled water using a high shear mixer (Silverson L4RT, Silverson Machines, Buckinghamshire, UK) at 2500 rpm for 10 min. The effects of pH and concentration of CMC were studied to obtain the best conditions for developing agar/CMC-based films. In this regard, different film-forming solutions (FFSs), containing 600 mg of agar and two different concentrations of CMC (10 and 40% *w*/*w* of agar), were prepared and their pH adjusted to 3 (acidic), 7 (neutral) and 10 (alkaline) using 1 N HCl or 1 N NaOH. The FFSs were then mixed for 20 min at 100 °C to obtain a homogeneous and clear mixture with a magnet stirrer. Further FFSs, all containing 600 mg of agar, were prepared at neutral pH and different concentrations of CMC (5, 10, 20, 40 and 60% *w*/*w* agar) to find the best ratio of agar/CMC blend, and by adding different concentrations of G-AgNPs (0, 0.5, 1 and 1.5% *w*/*w* of agar) to the optimised solution containing 40% CMC (*w*/*w* of agar) at neutral pH. The final volume of all the samples was 140 mL, containing 50% glycerol (*w*/*w* of agar).

All aforementioned FFSs were cast onto 15 cm diameter glass Petri dishes and dried in an oven at 35 °C for 24 h. Dried films were peeled from the casting surface and kept at 25 °C and 50% relative humidity before further characterisation.

### 2.3. Fourier Transform Infrared (FTIR) Spectroscopic Analysis

Sample functional groups were identified using an attenuated total reflectance–Fourier transform infrared (ATR-FTIR) spectrometer (Spectra Science Ltd., Chesham, Bucks, UK). The transmittance spectra were obtained at a resolution of 4 cm^−1^, from 4000 cm^−1^ to 650 cm^−1^ with 16 scans. The air was used as a reference for the background scan.

### 2.4. Morphological Analysis—Atomic Force Microscopy

The surface morphology of the agar/CMC-based film prepared in the presence or absence of AgNPs was analysed using an Atomic Force Microscope (NaioAFM, NanoSurf, Sedgefield, UK). Films were scanned over an area of 48 × 48 μm with a resolution of 256 (lines) × 256 (dots). The mean surface roughness (Ra) and the root mean square roughness (Rq) were collected from three different points on each sample.

The film’s surface microstructure was studied by scanning electron microscopy (SEM) (Zeiss Merlin FEG-SEM, Jena, Germany). The samples were fixed onto a silicon wafer, and images were taken at magnifications of 5.00 k × (2 µm) and 50.00 k × (200 nm). Images were edited using ImageJ 1.54 g software.

### 2.5. Optical Properties

The optical transmittance at wavelengths between 280 and 800 nm, as well as the ability to block ultraviolet (UV) radiation in the UVA (315–400 nm) and UVB (280–315 nm) ranges, were studied by using a Shimadzu UV-1900i spectrophotometer (Tokyo, Japan).

Ultraviolet (UV) radiation blocking was calculated based on the following formulas:

(1) $$UVA\;blocking\left(\%\right)=100-T_{UVA}$$

(2) $$UVB\;blocking\left(\%\right)=100-T_{UVB}$$

where T_UVA_ and T_UVB_ are the average transmittance values in the respective spectral ranges. Measurements were conducted in triplicate.

Film absorbance was assessed using the same spectrophotometer in the visible region (400–800 nm). Film transparency (T) was calculated by dividing the absorption of the film at 600 nm (A600) by its thickness in millimetres (L), using the following equation:

T = A600/L(3)

### 2.6. Mechanical Properties

The mechanical properties of the films were studied according to ASTM D882-02 [22] with a TA-XT2 texture analyser (Texture Technologies Corp., Hamilton, MA, USA). The film strips were cut to a dumbbell shape using the ASTM D412 [23] knife form, where the total length of the specimens was 125 mm, end width 16 mm, narrow part length 59 mm and narrow part width 6 mm. To measure elongation at break (EB) and tensile strength (TS), five specimens of each film were mounted between two callipers with a 10 cm initial length and a crosshead speed of 0.1 mm/s. A digital micrometer (IP54) with double measuring force was used to measure the film thickness at three random points for each film specimen.

### 2.7. Water Sensitivity

To measure the moisture content of the films, the samples were cut into pieces of 2 cm × 2 cm and dried in an oven at 105 °C for 24 h. Moisture content was calculated as the percentage change in weight of the film after oven drying, divided by the initial weight [24].

The solubility of the films was measured by placing the oven-dried film (W_i_) in 30 mL of water for 24 h at room temperature. Afterwards, the insoluble film samples were collected from the water and placed in an oven at 105 °C for 24 h (W_f_). The solubility is the percentage of the change in weight of the initial film and the final film divided by the weight of the initial film [25].

To study the swelling ratio, the weight (W_i_) of the film samples (4 cm × 4 cm) was recorded, then the films were immersed in 30 mL of water for 5 min and then collected; then, the surface was dried, and the weight (W_s_) of the samples was recorded. The swelling ratio was calculated as follows:

Swelling ratio = [(W_s_ − W_i_)/W_i_] × 100 (4)

The water vapour permeability (WVP) of the films was measured according to ASTM 1995 [26]. The films were cut into 2.5 cm diameter circles to cover cups containing 2 g anhydrous calcium chloride. Cups were placed in the desiccator containing saturated potassium sulphate at 25 °C, and weights were recorded daily for up to one week. WVP was calculated based on the following equation:

WVP = [∆w/∆t] × [X/A∆P](5)

The slope of the weight vs. time curve (Δw/Δt) equates to the water vapour transmission rate (g/s), X is the film thickness (m), A is the area of the film (m^2^) and ΔP is the partial water vapour pressure difference (Pa) between the two sides of the film.

### 2.8. Thermal Analysis

The thermal stability of the films was evaluated using a TGA Q50 Analyzer (TA Instruments, New Castle, DE, USA). Around 2 mg of each specimen was weighed into an aluminium pan and subjected to heating from 25 °C to 550 °C at a constant rate of 10 °C min^−1^ under a nitrogen atmosphere flowing at 40 mL min^−1^. The thermogravimetric data obtained were used to identify the maximum decomposition temperature (T_max_) from the corresponding derivative thermogravimetric curves.

### 2.9. Antimicrobial Activity

The antimicrobial activity of the films was measured by disc diffusion according to the method described by Al-Tayyar et al. [27]. Four bacterial strains were used, namely *Escherichia coli* ATCC 25922, *Listeria monocytogenes* 1043S, *Staphylococcus aureus* NCTC 8532 and *Pseudomonas aerujinosa* NCTC 10322. The bacteria were transferred to 3 mL of phosphate-buffered saline solution until the turbidity of the suspensions was approximately equal to that of a 0.5 McFarland standard (Fisher Scientific, Waltham, MA, USA), which is used as a turbidity standard, and corresponds to approximately 1.5 × 10^8^ CFU/mL. A sterile cotton swab from the bacterial suspension was spread on Müller–Hinton agar for all bacteria except *Listeria monocytogenes*, which was spread on tryptic soy agar. The films with different concentrations of AgNPs and the control film (no AgNPs) were punched to 6 mm diameter discs and placed on the previously swabbed plates and incubated for 24 h at 37 °C in an incubator. The inhibition zone is the clear area that appeared around the films and was free of bacterial colonisation [28].

### 2.10. Bio-Disintegration

The bio-disintegration of the agar/CMC blended films in the soil was studied using an adapted method by Mirpoor et al. [24]. The soil (Miracle-Gro houseplant potting mix, pH 6.3 ± 0.2, electrical conductivity 2.4 ± 0.6 dS/m) was poured into plastic trays (5 cm × 5 cm × 5 cm) up to a height of circa 3 cm, and the film pieces (1 cm × 1 cm) were buried in soil. The buried films were kept in an incubator set to room temperature (25 °C) for 30 days, and 1 mL of deionised water was sprayed on the top of the soil every 7 days. The weight of the films was recorded at different times (0, 1, 4, 7, 14, 30 days), and the weight residue (%) is calculated as the percentage of weight loss compared to the initial film weight. The experiment in simulated seawater (pH 7.8 ± 0.2) was performed in a similar way by soaking the films (1 cm × 1 cm) in 40 mL of seawater in 50 mL plastic tubes. The salt content of the seawater was recorded as 40 ± 5% (400 ± 50 ppm) using a salinity meter at a temperature of 25.2 ± 0.5 °C. Before weighing, samples were washed by soaking the pieces in 100 mL of deionised water for 10 s to remove the salts [29]. Weight loss was calculated as follows:

(6) $$Weight\;Residue\;\left(\%\right)=\frac{m0-mt}{m0}\;\times \;100$$

where m0 and mt are the weights of the corresponding film before (at time zero) and at each time point (1, 4, 7, 14 and 30 days), respectively.

### 2.11. Statistical Analysis

All experiments were performed in triplicate, and statistical analyses were conducted using IBM SPSS Statistics 27 software. One-way analysis of variance (ANOVA) was used to evaluate differences among treatments, followed by Duncan’s multiple range test at a significance level of *p* < 0.05.

## 3. Results and Discussion

### 3.1. Mechanical Properties of Agar/Cellulose Films

#### 3.1.1. Effect of Different pH and Cellulose Concentrations

**Figure 1:**
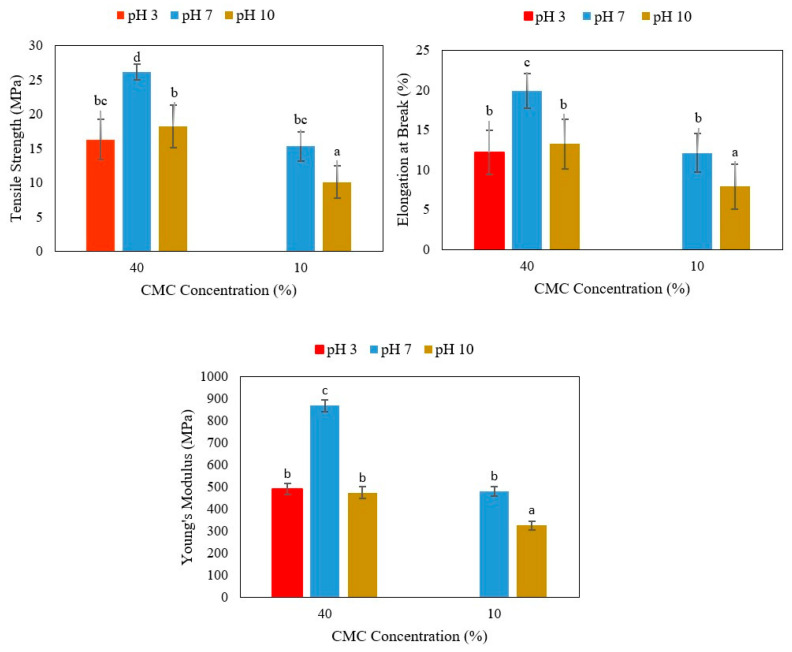
Mechanical properties of agar/CMC blend films at different CMC concentrations (10% and 40% *w*/*w* of agar) and pH values (3, 7 and 10). Different lowercase letters (a–d) indicate significant differences among the values reported in each graph (*p*  <  0.05).

The mechanical properties (tensile strength (TS), elongation at break (EB) and Young’s modulus (YM)) of the films prepared at three different pH values (3, 7 and 10) in the presence of 10% or 40% (*w*/*w* of agar) CMC are depicted in Figure 1. Firstly, at 40% (*w*/*w* of agar) CMC, the films prepared at both alkaline (pH 10) and acidic (pH 3) pH values displayed lower TS, EB and YM values than those prepared at neutral pH (7) and, therefore, show reduced elasticity and rigidity (Figure 1). Although at acidic pH values between 4 and 5 the agar can form elastic and transparent films, the film developed at acidic pH was not flexible, probably due to the extreme acidity [30]. Similarly, reduced elasticity and rigidity were observed for films prepared at alkaline pH (10) compared to neutral pH (7) using 10% (*w*/*w* of agar) CMC. It should be noted that no films were formed at acidic pH in the presence of 10% (*w*/*w* of agar) CMC.

**Figure 2:**
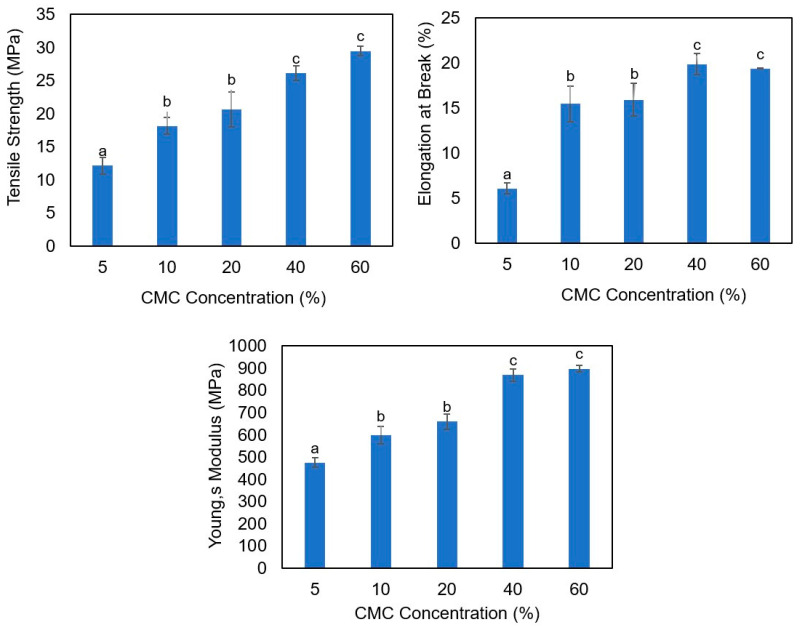
Mechanical properties of agar/CMC blended films at neutral pH and different CMC concentrations. Different lowercase letters (a–c) indicate significant differences among the values reported in each column (*p*  <  0.05).

The films prepared with different cellulose concentrations (5–60%) at neutral pH showed that the cellulose concentration significantly (*p* < 0.05) affects the mechanical properties of the bioplastic (Figure 2). By increasing the concentration of CMC, the films became stiffer and even more flexible due to the increase in the total mass of the polymers (the mass of the agar was kept constant in all the films, and it was 600 mg, while the mass of the CMC was increasing); the same results were reported by Mirpoor et al. [31] when they increased the mass of the cardoon protein. However, there was no significant (*p* < 0.05) difference between the bioplastics produced with 40% and 60% CMC; therefore, the films prepared at neutral pH in the presence of 40% CMC were selected for studying the effect of silver nanoparticles on the film’s characteristics.

#### 3.1.2. Effect of Different Concentrations of AgNPs

**Figure 3:**
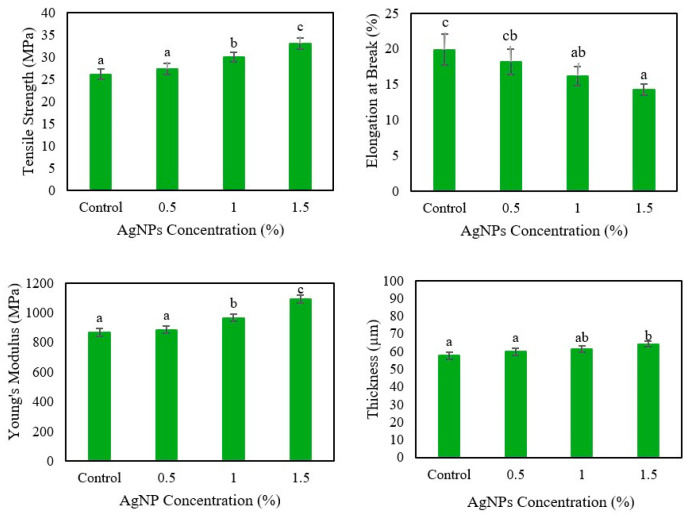
Mechanical properties of agar/CMC blended films containing different concentrations (0, 0.5, 1, 1.5% *w*/*w* of agar) of silver nanoparticles. Different lowercase letters (a–c) indicate significant differences among the values reported in each column (*p*  <  0.05).

The mechanical properties of the films are shown in Figure 3, and as can be seen, the mechanical properties were considerably affected by the addition of AgNPs. The thickness of the agar/CMC films slightly increased after the incorporation of silver nanoparticles into the matrix, which is probably related to the interactions between AgNPs and the polymers. The same results were reported by Shankar & Rhim [32] and Wardana & Widyaningsih [33], who developed films based on agar/lignin/silver nanoparticles and starch/agar edible films enriched with red cabbage, respectively.

With the addition of a higher concentration of silver nanoparticles, both the TS and YM of the film were increased, indicating a more resistant and rigid characteristic of the material. The dispersion of AgNPs in the matrix of the film controls the stress transfer at the interface between the matrix and the AgNPs, resulting in an increase in the tensile strength of the films [34]. Conversely, only a slight decrease in the EB of the films prepared with a higher concentration of AgNPs was observed. Thus, the flexibility of the film was only significantly (*p* < 0.05) affected in the agar/CMC-based film when 1.5% (*w*/*w* of agar) of AgNPs was added to the matrix. The reinforcement effect of AgNP addition on the film matrix can also be attributed to non-covalent interactions between the AgNPs and the hydroxyl groups of the biopolymer [35].

Similar reinforcing effects of the AgNPs on the film properties have been reported by Di Muzio et al. [36] and Sarwar et al. [37] for gellan gum nanocomposite thin films and PVA/nanocrystalline films containing silver nanoparticles, respectively.

### 3.2. Film FTIR Analysis

**Figure 4:**
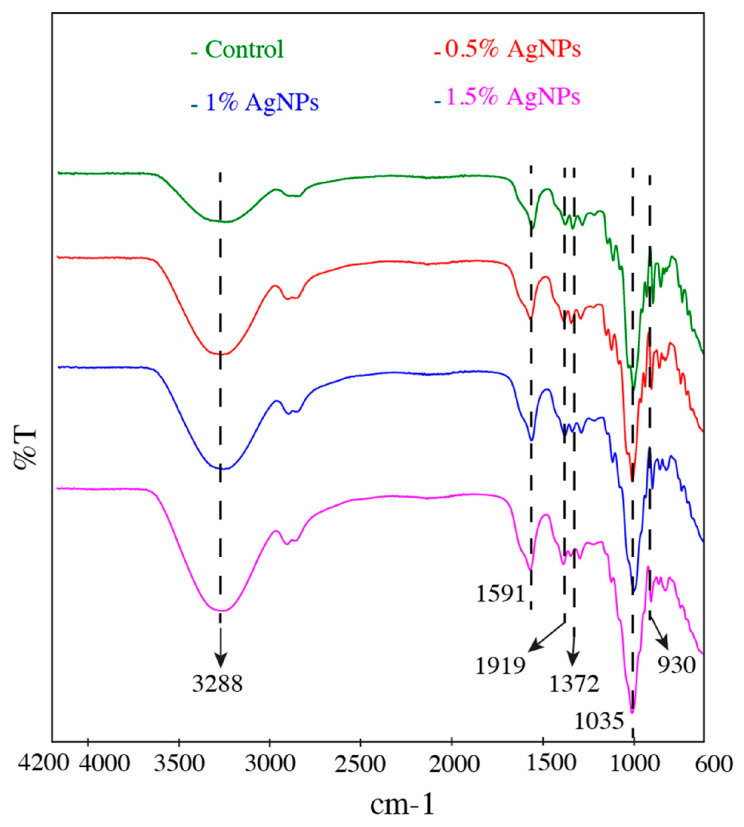
FTIR spectra of agar/CMC blended films containing different concentrations (0, 0.5, 1, 1.5% *w*/*w* of agar) of silver nanoparticles.

Figure 4 depicts the FTIR spectra of the developed films. The broad peak observed in all films at around 3288 cm^−1^ and 1372 cm^−1^ is due to the O-H stretching vibration and the ester sulphate group of the agar, respectively [9,38]. The characteristic peak of agar 3,6-anhydro-D-galactose at 1035 and 930 cm^−1^ is due to C=O stretching [39]. The asymmetric stretching vibration peak of the carboxyl group of the CMC appeared at 1591 cm^−1^, whereas the symmetric stretching vibration peak appeared at 1419 cm^−1^ [40]. The films containing AgNPs did not show any new peaks compared to the control film, and only some minor changes in peak intensity were observed; these could be due to the interactions between the biopolymers and the functional group (–COOH or –OH) of the AgNPs through van der Waals forces and hydrogen bonding [41]. The functional groups present in the plant extracts used for the synthesis of AgNPs and their interaction with silver ions play an important role in the nature of the functional groups of the green-synthesised nanoparticles [42]. This result shows that the addition of silver nanoparticles did not affect the chemical structure of agar/CMC-based films. Similar results were reported by Roy and Rihm [9] for starch/agar films functionalised with green-synthesised silver nanoparticles. Changes in the peak intensity of gelatin/agar-based films loaded with cranberry extract and linalool-loaded nanoparticles were reported due to physical interactions such as van der Waals forces and hydrogen bonding between the biopolymers and phenolic and flavonoid groups of cranberry extract [43].

### 3.3. Morphological Properties of Agar/CMC Films

**Figure 5:**
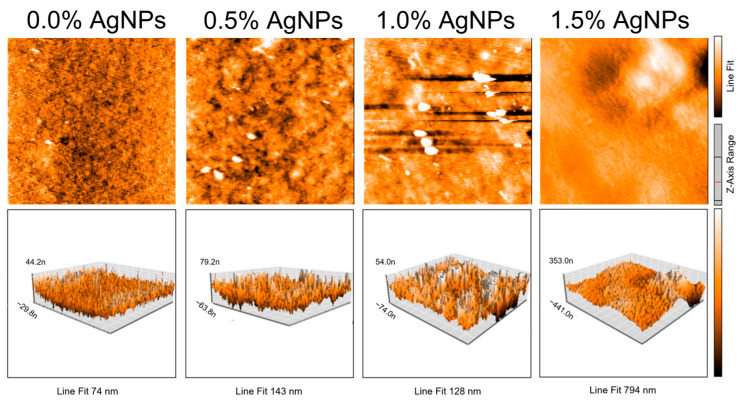
Atomic Force Microscopy images of agar/CMC blended films containing different concentrations (0, 0.5, 1, 1.5% *w*/*w* of agar) of silver nanoparticles. Films were scanned over an area of 48 × 48 μm with a resolution of 256 (lines) × 256 (dots).

The roughness of the films was measured using AFM. The average roughness (R_a_) and the root mean square roughness (R_q_) of the film samples, determined through AFM analysis, are reported in Table 1 and Figure 5. The results for both the R_a_ and R_q_ indicate a significant difference (*p* < 0.05) between the control and AgNP-loaded composite films. The control film exhibited a lower roughness value, indicating higher smoothness compared to the films containing AgNPs. By increasing AgNP concentration, film roughness increased, attributed to AgNP aggregation and uneven distribution within the matrix. Lower AgNP concentrations facilitate better dispersion across agar film surfaces, resulting in more uniform and smoother surfaces.

**Figure 6:**
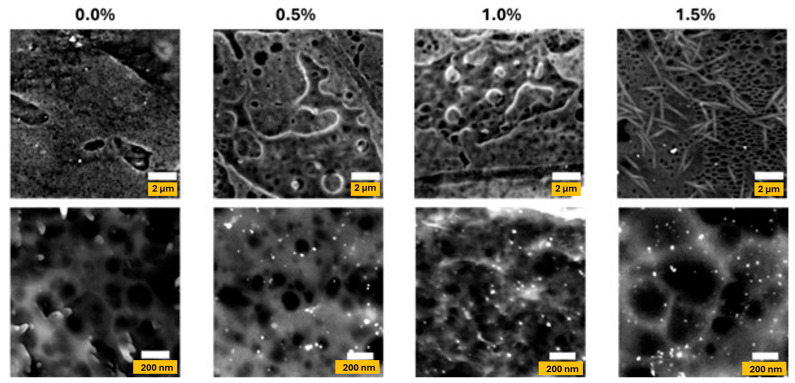
Scanning electron microscopy images of agar/CMC blended films containing different concentrations (0, 0.5, 1, 1.5% *w*/*w* of agar) of silver nanoparticles. The top row displays images captured using a magnification of 5 k × (2 µm scale). The bottom row shows images captured using a magnification of 50 k × (200 nm scale).

SEM images of the agar/cellulose films containing different AgNP concentrations (0–1.5%) are shown in Figure 6. The pores in the film matrix produced by the casting method are attributed to the solvent evaporation. There is a small reduction in pore size of the polymeric matrix of the film containing 0.5% (*w*/*w* of agar) AgNPs compared to the control film. Increasing AgNP concentration results in disruption of the porous network and larger pore sizes due to AgNP aggregation. In agar/CMC-based film when 1.5% (*w*/*w* of agar) of AgNPs were incorporated into the film matrix, the micro-structure is vastly different, with crystalline rods forming and a significant increase in pore size. These changes can influence the physical characteristics of the material, including gas permeability.

### 3.4. Film Optical Properties

**Figure 7:**
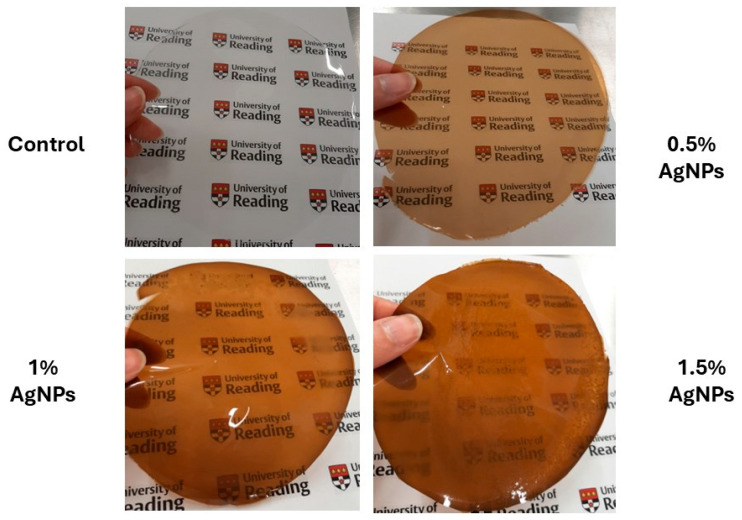
Images of agar/CMC blended films containing different concentrations (0, 0.5, 1, 1.5% *w*/*w* of agar) of silver nanoparticles.

The macroscopic images of the agar/CMC blended films containing different concentrations of silver nanoparticles are depicted in Figure 7. The control film is colourless and transparent, while the films containing AgNPs showed a pale orange colour.

**Figure 8:**
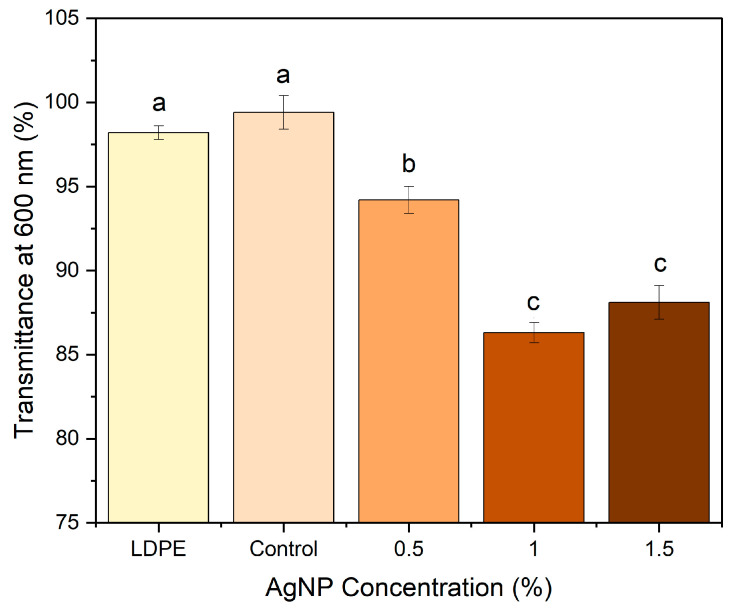
Comparison of transmittance values of agar/CMC blended films containing different concentrations (0, 0.5, 1, 1.5% *w*/*w* of agar) of silver nanoparticles at 600 nm. Different lowercase letters (a–c) indicate significant differences among the values reported in each column (*p*  <  0.05).

The transmittance of the films in the presence and absence of AgNPs was studied (Figure 8). The transparency and UV-shielding performance of the films were evaluated based on their transmittance in the visible and ultraviolet regions, respectively. LDPE film of similar thickness has been used as a comparable material. All films showed transmittance values above 80%, which is the minimum required to be considered transparent, and the transparency of the control film is similar to that of LDPE. Such a high transparency is due to the amorphous character of the agar polymer matrix.

**Figure 9:**
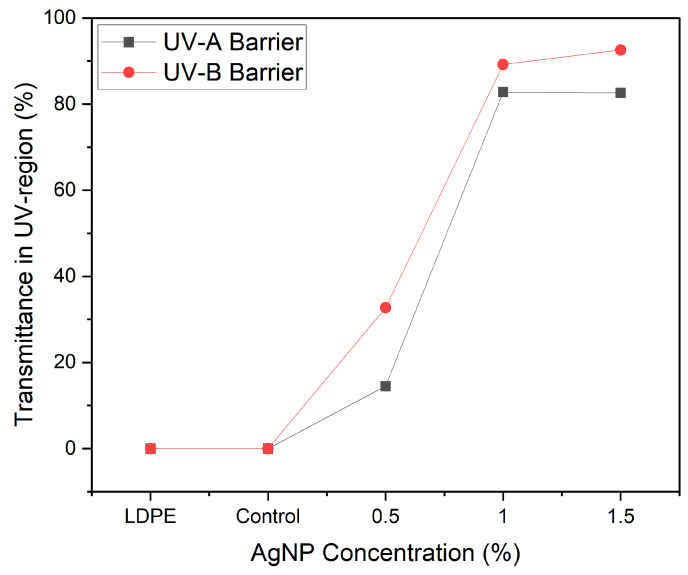
Ultraviolet (UV) radiation blocking of agar/CMC blended films containing different concentrations (0, 0.5, 1, 1.5% *w*/*w* of agar) of silver nanoparticles.

From transmittance spectra, the UV radiation blocking was calculated (*p* < 0.02) (Figure 9). This is an important parameter that can have a significant impact on food quality, as prolonged exposure to light, particularly ultraviolet light, can trigger photolysis and photo-oxidation reactions that affect the quality of the food. These reactions generate active oxygen and free radicals, resulting in the deterioration of food quality by causing unpleasant odours and altering its nutritional value.

The films’ UV-shielding ability was significantly improved by the incorporation of AgNPs across the UV wavelength range (230–400 nm). The control film did not demonstrate any UV-blocking capacity with values of ~0.2 and ~0.5% in the UV-A and UV-B, respectively. Integrating AgNPs into the film matrix increased the UV blocking of agar/CMC films to 14.49%, 82.82% and 82.62% in the UV-A region for films containing 0.5%, 1% and 1.5% AgNPs, respectively.

### 3.5. Water Sensitivity

One of the key challenges with hydrocolloid films in food packaging is their sensitivity to water [44]. The water sensitivity of the films was determined by evaluating different properties, including moisture content, swelling ratio, water solubility and measurement of water vapour permeability (WVP).

As shown in Table 2, the swelling ratio and water solubility of the films exhibited similar trends, decreasing when the concentration of AgNPs in the film matrix increased. The reduction in water solubility of films with a higher concentration of AgNPs could be attributed to the presence of insoluble silver nanoparticles. Dash et al. [45] reported that by increasing the concentration of AgNPs in a flaxseed protein-alginate film, the solubility decreased. Moreover, the high swelling ratio of the films, whether incorporated with silver nanoparticles or not, reveals the hydrophilic nature of the film. The reduction in the swelling ratio of films functionalised with silver nanoparticles could be due to the reduced interaction rate of the biopolymers with water molecules due to their interaction with silver nanoparticles [18,29].

The WVP of the films functionalised with AgNPs decreased compared to the control film up to 1 wt% of AgNPs in the film, followed by a slight increase with further increase in the AgNPs concentration to 1.5 wt% (Table 2). Several factors can affect the water permeability of the film, such as cross-linking, crystallinity, density and orientation of the different biopolymers used in the film matrix [46]. The decreased WVP of the films was mainly due to the increased tortuous path for water vapour diffusion by the impermeable nanoparticles distributed in the polymer matrix. However, the increase in WVP of the film with 1.5 wt% AgNPs was probably due to the aggregation of the nanoparticles at a higher concentration [19,47]. This result agrees with the results obtained by Roy et al. [34], who reported that the incorporation of AgNPs reduced the water vapour barrier properties of carrageenan films.

The films had a similar moisture content regardless of the presence and concentration of silver nanoparticles (Table 2). The moisture content of the films is primarily related to the number of free −OH groups and can be affected by the hydrophobicity of the filler [48]. Similar behaviour was previously reported by Sarwar et al. [37], who observed no significant difference between the polyvinyl alcohol/nanocrystalline cellulose-based films with different concentrations of silver nanoparticles.

### 3.6. Thermogravimetric Analysis

**Figure 10:**
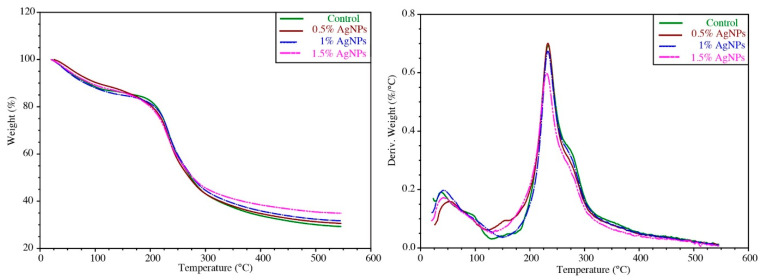
TGA thermograms of agar/CMC blended films containing different concentrations (0, 0.5, 1 and 1.5% *w*/*w* of agar) of silver nanoparticles.

The TGA thermograms of the agar/CMC and agar/CMC/AgNP composite films are shown in Figure 10. TGA curves indicate a weight loss in the films, while DTG curves show the maximum decomposition temperature. Multiple stages of thermal degradation were observed in all films. The initial weight change occurred at 60–110 °C, which was due to the evaporation of moisture in the composite films [49]. The subsequent thermal degradations were observed between 210 and 325 °C due to the decomposition of glycerol, agar and carboxymethylcellulose [50]. A similar thermal degradation pattern was observed for starch/agar-based films containing silver nanoparticles [9].

The weight residue of the agar/CMC film was 29.3%, which increased slightly in the agar/CMC films functionalised with the AgNPs (~35%), probably owing to the thermal stability of the AgNPs. This high residual weight content of the film can be related to the non-combustible minerals and impurities in the CMC and agar [51]. The same results were reported by Mahuwala et al. [51] and Roy & Rhim [49] about the high char content (>25%) of the film due to the non-combustible minerals and impurities. The DTG thermogram of the AgNP-loaded composite film showed similar behaviour to that of the control agar/CMC film, indicating that the thermal stability of the film was not affected by the addition of AgNPs.

### 3.7. Antimicrobial Activity

The disc diffusion method was used to evaluate the antibacterial activity of the films against four common foodborne pathogens. The results obtained for the control agar/CMC film and the films containing AgNPs are shown in Figure S1 and Table 3. The control film did not show any inhibitory effect against the four tested microorganisms. Depending on the concentration of AgNPs and the species of bacteria, the diffusion assay showed antimicrobial activity against both Gram-positive and Gram-negative bacteria. Dash et al. [45] reported that AgNPs prevent DNA replication and cell division.

Staphylococcal enterotoxins are common in high-protein foods like dairy products, meat and meat products and are produced by *S. aureus* strains, Gram-positive bacteria [52]. As can be seen in Figure S1 and Table 3, the agar/CMC films functionalised with 1.5 *w*/*w*% AgNPs revealed the antimicrobial activity against *S. aureus*. The same results were reported by Song et al. [38] for the gelatin/agar-based films in the presence of the highest concentration (8 wt%) of nanoparticles.

*E. coli*, the Gram-negative bacteria, are able to colonise the mucous membrane of the intestine and, as a result, cause a wide range of diseases in humans [53]. Similar results to *S. aureus* were obtained for *E. coli,* and only the film functionalised with the highest concentration of AgNPs (1.5 *w*/*w*%) had an inhibition zone around the film disc. Ortega et al. [54] reported the same effect of silver nanoparticles on *E. coli* for the starch films containing 143 ppm of silver nanoparticles, with a similar size of inhibition halo in this study.

*L. monocytogenes* is a Gram-positive bacterium that can grow in low-temperature conditions. It can therefore contaminate dairy products, ready-to-eat meals, raw milk and smoked seafood [55]. Although Gram-negative bacteria are more resistant to antimicrobial agents due to their hydrophobic cell wall, Gram-positive bacteria require lower concentrations of these agents to achieve considerable antimicrobial activity [56]. Figure S1 and Table 3 show that unlike the other bacteria, which showed antimicrobial activity only in the presence of 1.5 *w*/*w*% AgNPs, an inhibition halo against *L. monocytogenes* was also observed when the film contained 1 *w*/*w*% AgNPs.

*P. aeruginosa* is a Gram-negative bacterium that is resistant to several commonly used antibiotics and can grow on food stored at refrigerated temperatures [57]. The disc diffusion assay showed the antimicrobial activity for the film functionalised with 1.5 *w*/*w*% AgNPs.

The observed differences can be attributed to the structural characteristics of the films produced as well as to the amount of exposure of the active compounds incorporated in the matrix [58].

### 3.8. Films Bio-Disintegration

Bioplastic bio-disintegration in both seawater and soil is a fundamental aspect that determines material environmental impact, especially given the significant amounts of plastic waste that end up in marine environments [59] and that are disposed of within landfill sites. The introduction of bioplastics, designed to degrade more efficiently, offers a potential solution to mitigate these effects. However, the actual performance of these materials in natural environments needs careful evaluation.

Agar-based bioplastics are often highlighted for their promising biodegradability and low environmental impact. Studies show that agar-based bioplastics can break down effectively in both soil and marine environments, largely due to their composition and the presence of biodegradable polymers (e.g., polysaccharides) that are readily broken down by microorganisms [60].

#### 3.8.1. Soil Bio-Disintegration

**Figure 11:**
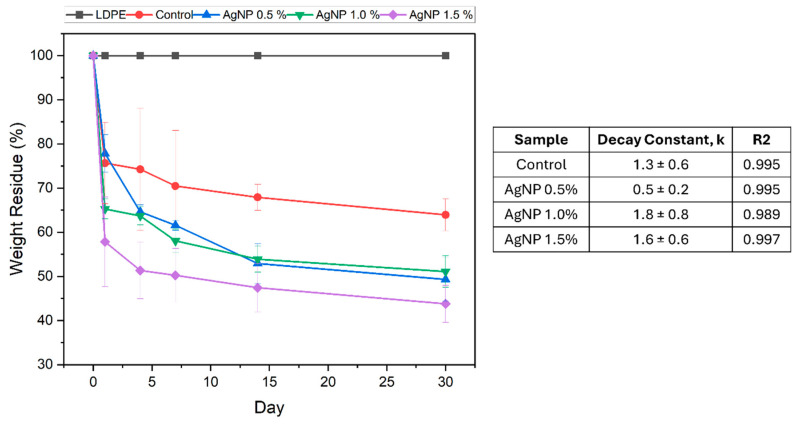
Percentage of agar/CMC blended films’ weight lost (**left**) and associated decay rate constants (**right**) in simulated soil conditions at different AgNPs concentrations. Curves were fit using an exponential decay function, y = A*exp(−kt), where y is the weight residue (%), A is the preexponential factor, k is the decay constant and t is the time (days).

The bio-disintegration profile of the films containing different concentrations of AgNPs was compared to that of the control and LDPE. The weight loss due to bio-disintegration in soil is presented in Figure 11, while the films’ visual appearances are reported in Table 4.

As expected, LDPE showed no disintegration in soil (Figure 11). Comparatively, the agar-based materials displayed significant weight loss, and interestingly, the presence of AgNPs facilitated the overall disintegration of materials in soil over the 30-day period (64% weight was retained for the control material, whereas 44–51% weight was retained for materials containing AgNPs). In all cases, the most significant weight loss occurred within the first 24 h. Within this period, the rate of disintegration increased from 26%/day (control) to 42%/day as the AgNP concentration increased to 1.5%. This initial loss in weight is most likely a result of water loss, which, as observed from the visual appearances of the materials (Table 3), facilitates bio-disintegration over the remaining time period. The decay constant at 0.5% AgNPs (0.5 ± 0.2 s^−1^) appeared to be lower than that of the control (1.3 ± 0.6 s^−1^), indicating a slower decay rate at low nanoparticle content. This behaviour could be attributed to enhanced water retention within the polymer matrix, resulting from interactions between water molecules and surface ligands on the AgNPs. However, the significant error in the decay constant for the control sample should be considered. At higher AgNP concentrations (1.0% and 1.5%), the decay constant was comparable to or exceeded that of the control. We attribute this to increased porosity in the polymer matrix, arising from the disruption of the polymer network at elevated nanoparticle loadings, enabling more efficient polymer degradation through microbial activity and hydrolysis. Additional factors may also contribute to this effect, including a greater solubility of the agar films in water and possible rearrangement of the polymeric matrix during drying, which could accelerate film degradation [61].

#### 3.8.2. Seawater Bio-Disintegration

**Figure 12:**
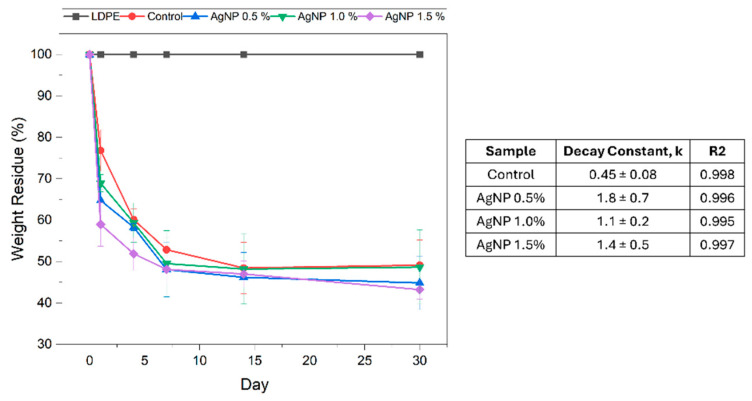
Percentage of agar/CMC blended films’ weight lost (**left**) and associated decay rate constants (**right**) in simulated seawater conditions at different AgNP concentrations. Curves were fit using an exponential decay function, y = A*exp(−kt), where y is the weight residue (%), A is the preexponential factor, k is the decay constant and t is the time (days).

The seawater bio-disintegration profile of the films containing different concentrations of AgNPs was compared to that of the control and LDPE. The results for films’ weight loss due to bio-disintegration in seawater are presented in Figure 12, while the films’ visual appearances are reported in Table 5.

Similarly to the results observed in soil, LDPE showed no signs of disintegration, whereas the agar-based materials exhibited significant weight loss over the 30-day testing period. In seawater the decay profiles of all agar-based films investigated were comparable, where the incorporation of AgNPs did not noticeably affect the overall weight loss over the 30-day period (Figure 11). However, relative to the control (0.45 ± 0.08 s^−1^), the decay constant increased in the presence of AgNPs (1.8 ± 0.7 s^−1^ at 0.5%) and remained largely unchanged with further increases in AgNP content. This suggests that the incorporation of AgNPs increases the rate of disintegration, likely due to the disruption of the polymer network, enabling a faster breakdown of polymer chains through hydrolysis and microbial activity.

It is worth noting that after 14 days of analysis, the films became brittle and prone to breakage upon drying, which may have influenced weight-loss measurements (Table 5).

Although AgNP incorporation did not alter the overall bio-disintegration behaviour in seawater, the combined findings from the soil and seawater tests highlight the strong potential of agar-based bioplastics as environmentally friendly materials with reduced persistence in natural environments.

## 4. Conclusions

Agar/CMC blended films containing various concentrations of green-synthesised AgNPs were obtained via solution casting. The incorporation of AgNPs imparted colour to the films while slightly reducing their transparency; however, all films remained transparent (over 80%). This also improved their UV protection, particularly in the films with the highest concentration of silver nanoparticles. The films developed in the presence of higher concentrations of AgNPs revealed a higher stiffness and lower elasticity, as well as enhanced hydrophobicity. The antimicrobial activity results confirmed that the films at the highest AgNPs concentration (1.5%) exhibited antimicrobial activity against the studied food-borne pathogenic bacteria. Moreover, the thermal stability of the films was not affected by the presence of AgNPs, which can be related to the unchanged chemical structure of the agar/CMC films as obtained from the FTIR spectra. The biodegradability rate of the films integrated with AgNPs, either in soil or simulated seawater, was around 50% after one month.

These results highlight the promising potential of the developed film containing 1.5% AgNPs as a biodegradable material with UV-blocking properties and optimal antimicrobial and mechanical properties. Such films could serve as an effective, environmentally friendly alternative to conventional plastics for food packaging applications, helping to extend product shelf life while reducing environmental impact.

Future studies should investigate oxygen and CO_2_ permeabilities, contact angles and AgNP migration.

## Figures and Tables

**Table 1:** AFM surface roughness of agar/CMC blended films containing different concentrations of silver nanoparticles. Different lowercase letters (a–g) indicate significant differences among the values reported in each column (*p*  <  0.05).

AgNP Concentration (%)	Roughness R_a_ (nm)	Roughness R_q_ (nm)
0	8.92 ± 1.24 ^a^	11.65 ± 2.29 ^e^
0.5	15.98 ± 2.07 ^b^	20.16 ± 3.07 ^f^
1.0	22.86 ± 3.81 ^c^	28.37 ± 5.20 ^g^
1.5	36.92 ± 4.26 ^d^	39.99 ± 14.24 ^g^

**Table 2:** Water sensitivity of agar/CMC blended films containing different concentrations of silver nanoparticles *.

AgNP Concentration (%)	WVP (×10^−9^ g m/m^2^ Pa s)	Swelling Ratio (%)	Moisture Content (%)	Solubility (%)
**Control**	1.24 ± 0.08 ^c^	1558.4 ± 69.48 ^c^	17.2 ± 0.81 ^a^	79.2 ± 3.48 ^c^
**0.5**	1.09 ± 0.05 ^ab^	1451.63 ± 75.69 ^bc^	16.74 ± 0.51 ^a^	75.1 ± 2.43 ^bc^
**1.0**	0.92 ± 0.06 ^a^	1338.8 ± 76.94 ^ab^	16.8 ± 0.92 ^a^	71.24 ± 1.84 ^ab^
**1.5**	0.98 ± 0.04 ^ab^	1228.9 ± 65.27 ^a^	17 ± 0.73 ^a^	67.33 ± 2.41 ^a^

* Different small letters (a–c) indicate significant differences among the values reported in each column (*p*  <  0.05).

**Table 3:** Inhibition zone showing the antimicrobial activities of the agar/CMC blended films containing different concentrations of AgNPs against different microbial pathogens. The unit for the reported inhibition zone is cm.

AgNPs Concentration (%)	Control	0.5	1.0	1.5
*Listeria monocytogenes 1043S*	No inhibition		0.097 ± 0.008 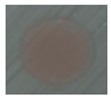	0.112 ± 0.005 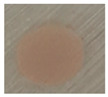
*Staphylococcus aureus NCTC 8532*	No inhibition			0.120 ± 0.012 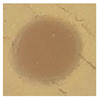
*Escherichia coli ATCC 25922*	No inhibition			0.136 ± 0.007 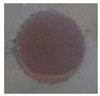
*Pseudomonas aerujinosa NCTC 10322*	No inhibition			0.105 ± 0.010 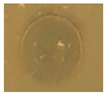

**Table 4:** Film appearance visual aspects at different AgNP concentrations in soil.

Sample	Day 1	Day 4	Day 14	Day 30
Control	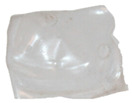	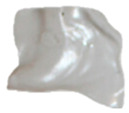	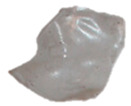	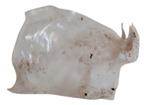
0.5% AgNPs	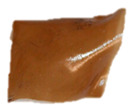	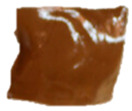	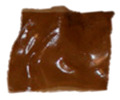	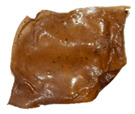
1.0% AgNPs	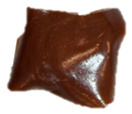	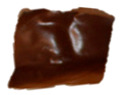	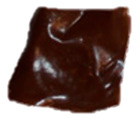	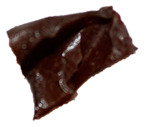
1.5% AgNPs	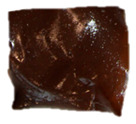	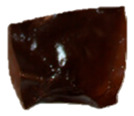	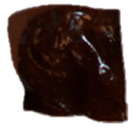	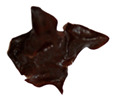

**Table 5:** Bionanocomposite visual aspect at different AgNPs concentrations in seawater.

Sample	Day 1	Day 4	Day 14	Day 30
Control	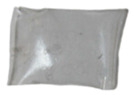	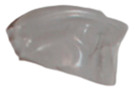	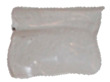	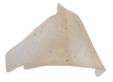
0.5% AgNPs	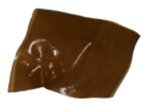	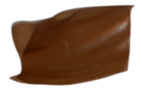	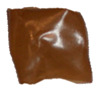	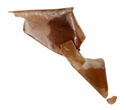
1.0% AgNPs	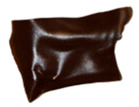	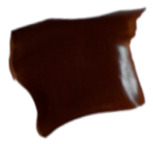	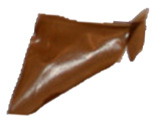	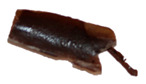
1.5% AgNPs	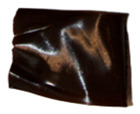	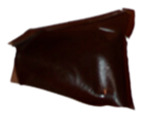	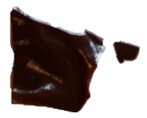	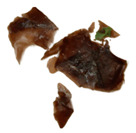

## Data Availability

The data presented in this study are available on request from the corresponding author due to confidentiality restrictions.

## Supplementary Materials

The following supporting information can be downloaded at: https://www.mdpi.com/article/10.3390/polym17233126/s1, Figure S1: Antimicrobial activities of the agar/CMC blended films containing different concentrations (0, 0.5, 1 and 1.5% *w*/*w* of agar) of silver nanoparticles.; Figure S2: Gas permeability of the agar/CMC blended films containing different concentrations (0, 0.5, 1 and 1.5% *w*/*w* of agar) of silver nanoparticles.
