# Supplementary material for: Agar/Carboxymethyl Cellulose Blended Films with Green-Synthesised Silver Nanoparticles as a Sustainable Alternative for Food Packaging Applications
Source: Polymers (Basel). 2025 Nov 25;17(23):3126. doi: 10.3390/polym17233126 (PMC12694301; doi:10.3390/polym17233126)
Supplement: Supplementary file 1 [file polymers-17-03126-s001.zip › polymers-3970112-supplementary.pdf]

Article

Agar/Carboxymethyl Cellulose Blended Films with Green-Synthesised Silver Nanoparticles as a Sustainable Alternative for Food Packaging Applications

Seyedeh Fatemeh Mirpoor [1,*], Alessio Massironi [2], Danielle Winning [2], Stella Lignou [1], Sameer Khalil Ghawi [1], Federico Trotta [2] and Dimitris Charalampopoulos [1]

[1] Department of Food and Nutritional Sciences, University of Reading, Harry Nursten Building, Pepper Lane, Whiteknights, Reading RG6 6DZ, UK
[2] Metalchemy Limited, 71-75 Shelton Street, London WC2H 9JQ, UK
* Correspondence: s.mirpoor@reading.ac.uk

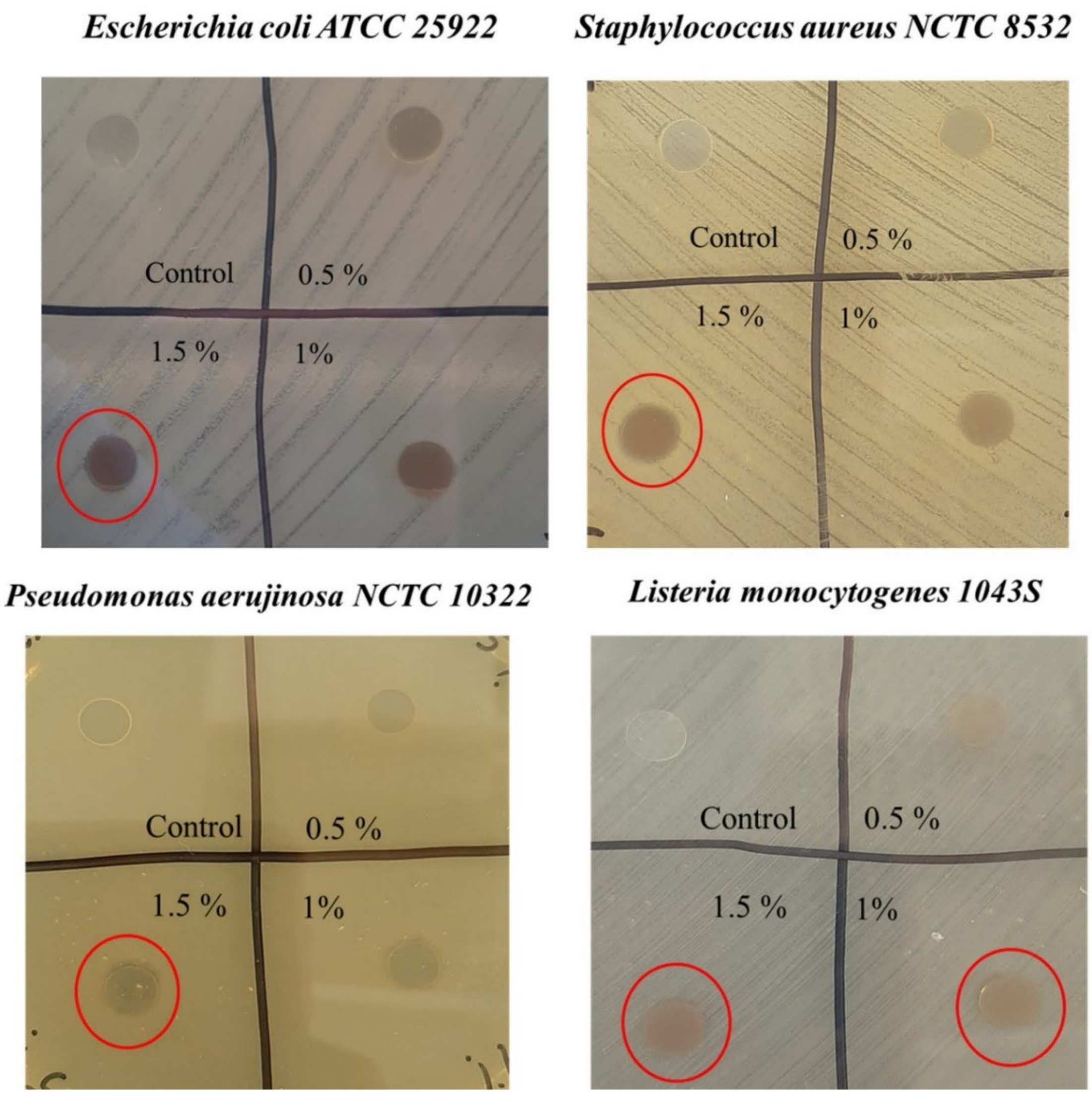

Figure S1. Antimicrobial activities of the agar/CMC blended films containing different concentrations (0, 0.5, 1 and 1.5% w/w of agar) of silver nanoparticles.

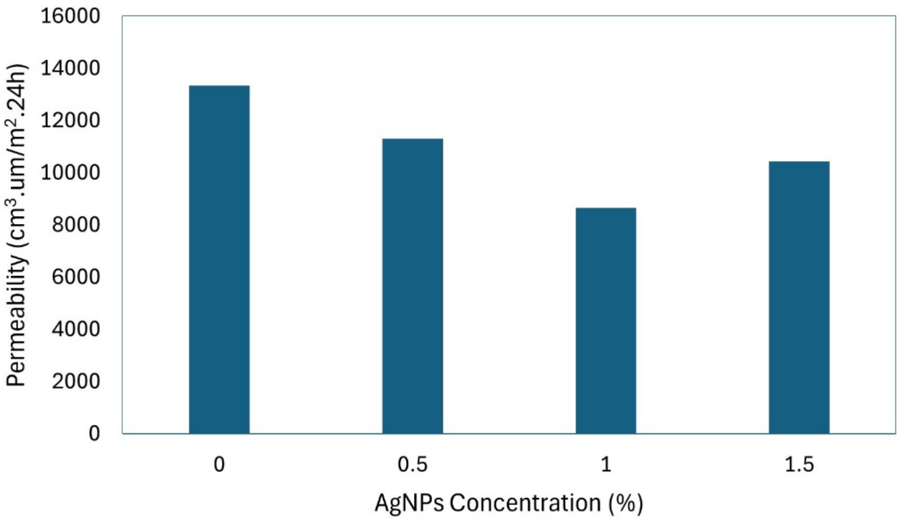

Figure S2. Gas permeability of the agar/CMC blended films containing different concentrations (0, 0.5, 1 and 1.5% w/w of agar) of silver nanoparticles
